# Effect of Educational Program on Knowledge, Attitude, and Willingness of Nursing Students for Hematopoietic Stem-Cell Donation

**DOI:** 10.3390/ijerph16193696

**Published:** 2019-10-01

**Authors:** Miok Kim, Minho Shin

**Affiliations:** 1 College of Nursing, Dankook University, Cheonan 31116, Korea; 2 Department of Computer Engineering, Myongji University, Yongin 17058, Korea

**Keywords:** hematopoietic stem cell, donation, knowledge, attitude

## Abstract

This study explored how an educational program on hematopoietic stem-cell donation (HSCD) affects the knowledge, attitude, and willingness for HSCD among nursing students. The subjects were the nursing students at a university in Korea: 43 in the experimental group and 42 in the control group. All subjects took a pre-test, and only the experimental group attended an educational program. Both the groups completed two post-tests. Variables of interest were knowledge, attitude, willingness, and registration ratio for HSCD. The educational program increased knowledge (*F* = 8.093, *p* < 0.001) and attitude (*F* = −6.422, *p* < 0.001) of the experiment group. After the program, the experimental group showed higher willingness for HSCD (*χ^2^* = 7.609, *p* = 0.006) and higher registration ratio for HSCD (*χ* *^2^*= 4.258, *p* = 0.039) compared to the control group. The educational programs for knowledge and attitude about HSCD will affect the students’ future nursing, and influence clients and their families toward positive perception on HSCD and organ donations.

## 1. Introduction

Hematopoietic stem cells (HSC) can produce blood cells such as leukocytes, red blood cells, and platelets through proliferation and differentiation in bone marrow, peripheral blood, and umbilical cord blood. Hematopoietic stem-cell transplantation (HSCT) is an effective treatment for blood and immune-related diseases and some hereditary metabolic diseases. HSC can be transplanted through the use of blood from non-hematological donation if the human leucocyte antigen (HLA) matches. The best donor for HSCT is an HLA-matched sibling or unrelated donor. However, with less than 30% probability, patients can have a matched sibling donor [1].

Kekre and Antin [2] stated that, as the population of Europe and North America becomes more diverse, aggressive strategies need to be sought for finding matched unrelated donors.

While the annual cumulative number of potential HSCT recipients reached 3000 from 2013 to 2015, fewer than 100 patients received the HSCT from unrelated donors every year [3]. To increase the donors-to-patients ratio, not only are more efforts to promote donations needed, but we also need to provide systematic education for potential donors to have the right knowledge and confidence about HSCT.

The unrelated hematopoietic stem-cell transplantation donation (HSCD) initiative aimed to recruit 300,000 donors by 2015 [4]. Because of the polymorphism of HLA, however, 200,000 donors are required for the HLA match ratio of 80%, and 400,000 donors for the ratio of 90% [5]. Beom, Kim, Kim, and Kim [6] argued that the number of pledgers should be at least 500,000 to increase HLA concordance and make the HSC treatment effective.

Even if the HLA matches, only 57% of the registrants made actual donations. This was lower than the 70–80% in developed countries [7]. According to Oh [8], the reasons for their withdrawal included self-rejection (57.8%), opposition from family members (41.1%), and other reasons (1.0%). To transform the registrants’ will for HSCD into actual donation, education programs and campaigns should be provided throughout the society.

Any healthy person of age from 20 to 40 can register for HSCD. The donations from young donors tend to show successful results [9,10], and they are more likely to keep their will for HSCD for a long time of period. Therefore, securing donations from young people is critical for the wide success of HSCD [11].

To encourage HSCD, developing appropriate knowledge and attitude regarding HSCD among healthcare providers and establishing a professional counseling system should be prioritized. Galen [12] found that the knowledge and attitude of donors, such as organ transplant coordinators, had a positive impact on the promotion of organ donation. Cebeci, Sucu, and Karazeybek [13] showed that the healthcare provider’s professional counseling for recruiting donors for HSCD helped communicate the right knowledge to them, leading to the desirable decisions for their donations. Positive awareness and correct information about organ donation among nurses can promote donor’s will and self-esteem for donation, and help the recipients and their family to promote public awareness of organ donation.

This study aimed to develop an educational program about HSCD for nursing students, and identify its effect on their knowledge, attitude, and willingness for HSCD. The hypotheses of this study were as follows:

(1) The experimental group that participates in the educational program will have higher knowledge scores on HSCD than the control group.
(2) The experimental group that participates in the educational program will have higher attitude scores on HSCD than the control group.
(3) The experimental group that participates in the educational program will have higher rates of willingness for HSCD than the control group.

## 2. Materials and Methods

This study used a non-equivalent control group pre- and post-test design.

### 2.1. Study Subjects and Sampling

The inclusion criteria were as follows: nursing students who and whose family members did not experience chronic illness and blood-related diseases, and who understood the purpose of the study and decided their participation voluntarily. Exclusion criteria were as follows: nursing students who and whose family members did experience chronic illness or blood-related diseases.

The sample size was determined using the G*Power 3.10 (Heinrich-Heine-Universität Düsseldorf, Düsseldorf, Germany) program. The sample size for comparing willingness for HSCD in two groups was calculated based on a significance level (*α*) of 0.05, a power (1−*β*) of 80%, and a medium effect size (*w*); a sample size of 54 for each group was calculated, having 108 in total.

A study by Kaya et al. [14] applied educational programs to improve knowledge and attitude of first-year students about HSCT and HSCD with a large effect size (*w*) of 0.65. While their study evaluated the effect on the willingness to donate stem cells immediately after a brief lecture, this study evaluated the effect on the subject’s donor registration for 12 weeks after the education program. Therefore, we used a medium effect size.

Starting with 103 subjects in the pre-test, 85 subjects finished the program and post-test; out of 53 subjects in the experimental group, three left the school, two failed to complete the test, and five failed to meet the necessary experiments (dropout rate of 23.2%); out of 50 subjects in the control group, five did not complete the test and three withdrew (dropout rate of 19.0%). There was no statistically significant difference in the demographic characteristics and disease characteristics between the two groups.

As the actual sample size was less than the designed sample size, a post-hoc test was conducted based on the results of this study in order to examine the appropriateness of the assumption of the intermediate effect size and the number of participants in the actual study. As a result, the medium effect size of the knowledge about HSC was 1.73, the attitude was 1.58, and the power was 1.00 and 0.99 for knowledge and attitude, respectively. We confirmed that we had the appropriate number of samples to meet the purpose of the study.

### 2.2. Research Instruments

*Knowledge about HSCD*. Knowledge about HSCD was measured using the HSC knowledge scale developed by Kim and Ahn [15]. The instrument consists of 20 items with responses of “yes”, “no”, and “do not know”. A correct answer is scored 1, and wrong and “do not know” responses are scored 0. The internal consistency of the items using KR-20 was 0.83 in Kim and Ahn [15] and 0.82 in this study.

*Attitudes regarding HSCD*. A scale for the attitude regarding cord blood was developed by Kim and Ahn [15], and we modified this scale for measuring the attitudes regarding HSCD. It consists of 10 items in a four-point Likert scale (1: definitely disagree, 2: disagree, 3: agree, 4: definitely agree). Higher scores on this scale indicated better attitudes toward HSCD. Cronbach’s alpha was 0.84.

*Willingness for HSCD.* The intention for HSCD was measured after two weeks and 14 weeks since the education program ended. In the first post-test, willingness for donation was assessed by “yes” or “no”. The second post-test assessed the ratio of registration for HSCD.

*General characteristics.* The general characteristics of subjects were measured using 12 items including sex, age, experience of blood donation, experience of registration for brain organ donation, recognition of registration process of HSCD, acknowledgement of recovery process after HSCD, and exposure to promotion or campaign for HSCD.

### 2.3. Development of the Program

The program was drafted through the analysis of the present status and achievements of the domestic and foreign initiatives for HSCD registrations, the study of reports about HSCD registrations from the related organizations, and the literature survey including the Bone Marrow Transplant Team at Hamilton Health Sciences of Juravinski hospital and cancer center [16]. The final program was developed by three HSCD experts: two nursing professors and one medical professor.

The education program consisted of four sessions. The first session provided lectures about human, society, and life. The second session introduced diseases needing organ donations, donors, and the status and outcome of domestic and foreign movements for organ donation. The third session discussed the mission for the donation-based life-savings in terms of individuals, family, and society. The fourth session was constructed to create awareness about the value of donating organs by exploring the meaning of life, and ultimately to induce mindfulness to participate in organ donation. Each session included group discussions and presentations to share the beliefs and values of the members. At the end of each session, participants wrote a short memo about their thoughts and beliefs experienced through the sessions, and hung it on a tree to review and confirm their attitude. The program was conducted twice a week, and each session was 2 h, taking two weeks for the whole program.

The contents of the program are explained in detail in Table 1.

### 2.4. Data Collection

This study was approved by a Bioethics Review Committee (1041479-201609-HR-022). All the subjects were informed about their rights and anonymity, and we received their informed consent to participate in the study. For ethical consideration, the control group was given an opportunity to receive HSC education after a post-test.

**Figure 1:**
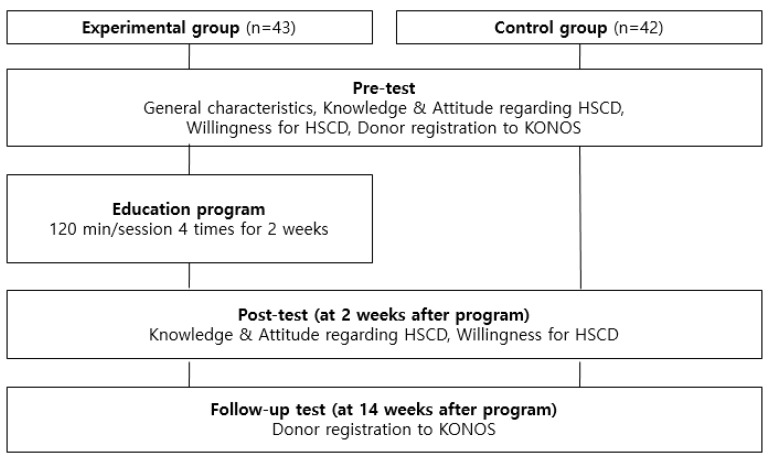
Flow of the study (HSCD: Hematopoietic Stem-Cell Donation, KONOS: Korean Network for Organ Sharing).

This study was conducted from September 2016 to March 2017. The first voluntary participants were assigned to the control group. After the first pre-test was conducted, the first post-test was taken in four weeks, and the second post-test was performed 12 weeks after the first post-test. After the first post-test of the control group, the experimental group was recruited. After a pre-test followed by a two-week program, the first post-test was given to the experimental group two weeks after the program. The second post-test was taken 12 weeks after the first post-test. Figure 1 illustrates the procedure of the program in detail.

*Pre-test.* Self-report questionnaires were used to measure the general characteristics of the subject; knowledge, attitudes, and willingness regarding HSCD; and HSCD registration. The pre-test of the control group was given in September 2016 and that of the experimental group was given in November 2016.

*Application of the program.* The educational program was given to the experimental group for two weeks, two sessions per week, in November 2016. Each session consisted of an introduction for 10 min, a lecture for 70 min, group discussion and presentations for 30 min, and closing for 10 min. When closing, each participant made notes about their thoughts and posted on their board, so that they could review them in later sessions. Each session was given in the same place throughout the program for consistency. Questions or comments were made to the researchers in person or via short message service (SMS).

*Post-test.* The post-tests for the experimental group were conducted two weeks and 14 weeks after the program, while the post-tests for the control group were conducted four weeks and 16 weeks after the pre-test. Each post-test took 15 min. The first post-test measured the knowledge, attitude, and willingness of the participants for HSCD. The second post-test measured the ratio of HSCD registration of the participants.

### 2.5. Data Analysis

The collected data were statistically analyzed using SPSS WIN 25.0 (IBM Inc. Armonk, NY, USA). The normality assumption of the dependent variable was tested using the histogram, the degree, and the Kolmogorov–Smirnov (K–S) test. As a result, the knowledge about HSCD satisfied the normality assumption (D range: 0.09–0.11, *p* > 0.05).

Skewness and kurtosis satisfied the normality assumption because there were no variables exceeding the critical value of ±1.96 at the 0.05 significance level [17]. Therefore, it was analyzed using the parametric statistics. Conversely, the attitudes toward HSCD did not satisfy the K–S test (D range: 0.12–0.15, *p* < 0.05), and it was analyzed using the non-parametric statistical method.

(1) The *χ^2^* test was used for assessing homogeneity between the experimental group and the control group.
(2) The homogeneity test of the knowledge and attitude regarding HSCD in the experimental group and the control group was analyzed using an independent *t*-test and Mann–Whitney U test.
(3) The difference in knowledge and attitude regarding HSCD between the experimental group and the control group after the educational program was analyzed using the mean, standard deviation, and analysis of covariance (ANCOVA).
(4) After the educational program, the difference in HSCD registrations between the experimental group and the control group after two weeks of intervention and 12 weeks after the intervention was analyzed using the *χ^2^* test.

## 3. Results

### 3.1. General Characteristics and Homogeneity of Subjects

Table 2 shows the results of the homogeneity test for the general characteristics of all participants. There was no difference in general characteristics between the two groups

The knowledge and attitudes regarding HSCD showed no significant difference between the two groups (Table 3).

### 3.2. Comparison of Knowledge Regarding HSCD

To measure the change in knowledge before and after the program, the covariate was used as the pre-test value and tested with ANCOVA. The HSCD knowledge score was significantly higher in the experimental group (0.76) than in the control group (0.35), supporting Hypothesis 1 that “experimental group undergoing the program will have higher knowledge about HSCD than the control group” (Table 4).

### 3.3. Comparison of Attitudes Regarding HSCD

Pre-test values of attitude were used as covariates and analyzed using ANCOVA. As a result, post-test scores of the attitudes regarding HSCD showed statistically significant differences between the experimental group that scored 3.86 ± 0.37 points and the control group that scored 3.12 ± 0.35 points. Hypothesis 2, i.e., “the experimental group with the program will have a higher score on the attitude about HSCD than the control group without the program”, was supported (Table 4).

### 3.4. Comparison of the Willingness for HSCD

The intention to engage in HSCD was measured three times; willingness for HSCD was measured in the pre-test and the first post-test, and the registration to Korean Network for Organ Sharing (KONOS) was checked in the second post-test.

In the pre-test, there was no significant difference in the willingness for HSCD between the two groups. The results of the first post-test showed that there was a significant difference between the experimental group and the control group (*χ^2^* = 7.609, *p* = 0.006) (Table 5). There was also a significant difference between the experimental group and the control group in the second post-test (*χ^2^* = 4.258, *p* = 0.039) (Table 6).

Therefore, Hypothesis 3, i.e., “experimental group that underwent the educational program would have more willingness for HSCD than the control group”, was supported.

## 4. Discussion

There was no significant difference between the two groups in the pre-test score in terms of the knowledge about HSCD. This is similar to the findings of Kim and Ahn [15]. In the post-tests, however, the knowledge of the experimental group was significantly higher than that of the control group. This is the same as the findings by Kaya et al. [14], which reported that the knowledge and awareness of HSCT improved after the first-year HSCT students in medical school. Based on the findings of Kim and Ahn [15] and Kwok et al. [18] that higher knowledge level leads to higher level of willingness for HSCD, it is expected that the promotion of knowledge about the benefit of HSC, diseases requiring HSCT, the HSCD registration procedure, and the procedure and methodologies of HSCT can also improve the willingness for HSCD.

Although there are no serious adverse events in bone marrow (BM) and peripheral blood stem cell (PBSC) donations [19], the donor’s safety is the most important issue. Donor’s health is rarely threatened because the donor’s blood levels are constantly monitored and controlled until the blood level returns to normal after donation. Nevertheless, misconceptions about HSCD exist, such as “HSCD hurts”, “can be done only within a family”, and “paralysis of the spine is possible”. It is necessary to provide the right information because there are many misconceptions about HSCD.

HSCT is a complex procedure requiring comprehensive education of patients, donors, and caregivers. A positive knowledge of HSCT and HSCD among healthcare providers could increase unrelated donors [20]. It was reported that misconceptions about HSCD among medical students may interfere with participation in the bone marrow registry (BMR) [21]. Therefore, the expansion of the medical education curriculum can help recruit and retain donors by improving the knowledge gap in BMR and HSCT for the next generation of healthcare providers [21]. Nurses should try to ensure that potential donors have the right information and knowledge about the efficacy and safety of the procedures and methods for donation and transplants.

For attitudes about HSCD in the pre-test, there was no significant difference between the experimental and control group. This was similar to the study by Kim and Ahn [15]. The attitude scores of the experimental group were significantly higher than that of the control group after the program. This shows that the educational program for HSCD promotion was effective in improving the attitude toward HSCD. The HSCT education provided for medical students in the study by Kaya et al. [14] led to more positive attitudes toward HSCD.

Morgan et al. [22] suggested that television promotions can help encourage organ donation, and emphasized the importance of public education for social perception. The in-depth interview and observation of 10 liver transplantation donors identified the motivation for liver donation, lack of information, lack of doctor’s management, physical difficulties, psychological and emotional ambivalence, social and economic difficulties, desire, and overcoming difficulties [23]. Providing correct knowledge using appropriate media and promoting attitudes through continuous public relations and education should be given priority. Healthcare professionals should understand the donor’s emotions and work together to minimize their physical and psychological difficulties.

In the pre-tests, 62.8% and 64.3% of those in the experimental and control groups, respectively, had intentions for HSCD. This was similar to the findings of the prior study [15], where 63.8% of nursing students showed willingness for HSCD. In the first post-test, 95.3% and 73.8% of the experimental and control groups, respectively, showed intentions to donate, indicating a significant difference between the two groups. These results suggest a positive contribution of the education program to the improvement of the subject’s intention for HSCD.

According to a survey on the awareness of organ donation by the Ministry of Health and Welfare in Korea, 40.6% of people were willing to donate blood and 29.2% to donate organs, while only 14.3% were willing for HSCD. The reluctance for HSCD is mainly due to unclear fear (organ donation 46.2%, blood donation 36.4%, and HSCD 52.3%) and fear of body damage (organ donation 36.9%, HSCD 26.5%). In this study, the intention for HSCD in the experimental group significantly increased after the education program, by improving their knowledge and attitude and eliminating relevant fears.

In the second post-test, 23.3% and 7.1% of the experimental and control groups, respectively, registered as donors, confirming a positive effect of the educational program on increasing HSCD. The more registered donors exist, the higher the probability of finding a matching donor is; thus, the increase in registrations is an essential requirement for HSCD promotion.

For the past 20 years, only 58.1% of donation registrants from one Korean HSCD registry agreed to donate when they were requested for an actual donation [24]. This agrees with the reports by Denzen et al. [20] and Passweg et al. [25] that there is a gap between needs and donation of unrelated HSCT in many developing countries.

As the HSCD registration increases, retaining the members becomes crucial [26]. Kollman et al. [9] reported that, in the National Marrow Donor Program, which is an unrelated HSCD registrar in the United States, improving the donor retention rate by 1% is equivalent to recruiting 28,000 new donors, and reported that it is cost-effective to retain the donor’s willingness to donate.

Continual communication with registered donors is crucial to maintain HSCD participation [27]. Effective follow-up management systems are needed to help registrants secure information to retain favorable beliefs about donation [6]. Additionally, it is necessary to have the government’s aggressive policies and budget, efficient reorganization of donation registration and donation programs, development of education programs related to HSCD, and systematization of follow-up programs for donors.

This study has limitations in its generalizability of the results, as the survey was conducted with nursing students from one university. However, the results can be used to facilitate recruitment of donors and management of donations by registrants and organ donation movements by college students for promoting HSCD.

The nursing students, trained by educational programs designed for promoting knowledge and attitude for HSCD, will positively affect their future nursing, and influence their clients and clients’ families toward positive perception on HSCD and organ donations.

The beliefs and attitudes of nursing college students can affect their nursing, clients, and clients’ families in future. Nursing students should understand diseases that require organ donation and help related subjects such as HSCD donors and recipients to establish a positive perception on HSCD and organ donations. Therefore, within the college curriculum, it is necessary to provide a systematic education designed for promoting knowledge and attitude about HSCD.

## 5. Conclusions

This study aimed to develop an educational program for HSCD activation and identify the effects of program on the knowledge, attitude, and willingness regarding HSCD for nursing students. After the program, their intention for future donation was measured after two weeks, and then their actual registration for HSCD was checked after 14 weeks. As a result, knowledge, attitude, willingness for HSCD, and the registration ratio of the students in the program were significantly higher than those not in the program.

Within the college curriculum, it is necessary to include systematic education, including the value and realization of organ donation, such as HSCD. Additionally, we suggest identifying the decision process the donor experiences from the registration to the donation, improving the potential donor management system accordingly.

## Figures and Tables

**Table 1:** Contents of the education program for hematopoietic stem-cell donation (HSCD).

Session	Topic	Content
1st	Human and Society	Dignity and value of human and their social roles
	Life	What kind of life we make Story of blessed life, people with enriched life (voices of donors and recipients)
	Team discussion and presentations	Note and questions
	Planting hope tree	
2nd	Thinking about donating stem cells?	Questions about donating stem cells What are stem cells? How are stem cells collected? Will I be accepted as a donor? Donating peripheral blood stem cells What happens before and during donation? Growth factor injections and related risks Apheresis and related risks What are the risks for female donors?
	Diseases requiring HSCD and management of them	Status (Donor-to-recipient ratio, examples), collecting, transplant, and recovery of HSCD
	Organ donation campaign results, domestic and foreign	Websites related to organ donation campaigns
	Team discussion and presentation	Note and questions
	Planting hope trees	
3rd	Future mission for organ donation	Future challenge for organ donation: the individual, family, and social aspects
	Team discussion and presentation	Note and questions
	Planting hope trees	
4th	Life goal and vision	Life goal setting and sharing life vision
	Team discussion and presentation	Note and questions
	Planting hope trees	

**Table 2:** Homogeneity test of the two groups on general characteristics.

Characteristics	Categories	Exp. (*n* = 43) *n* (%)/Mean ± SD	Con. (*n* = 42) *n* (%)/Mean ± SD	*χ^2^ (p)*
Sex	Male Female	14 (32.6) 29 (67.4)	7 (16.7) 35 (83.8)	2.884 (0.089)
Age (years)		20.20 ± 1.20	19.92 ± 0.83	1.243 (0.217)
Clinical practice experience	Yes No	12 (27.9) 31 (72.1)	7 (17.1) 34 (82.9)	1.407 (0.235)
Blood donation experience	Yes No	27 (62.8) 16 (37.2)	22 (52.4) 20 (47.6)	0.943 (0.332)
Number of blood donation (*n* = 49)	1–5	6 (25.0)	4 (19.0)	3.036
	6–10	2 (8.3)	3 (14.3)	(0.552)
	11–15	8 (33.3)	10 (47.6)	
	16–20	6 (25.0)	4 (19.0)	
	Above 21	2 (8.3)	0 (0.0)	
Family with chronic disease	Yes No	21 (48.8) 22 (51.2)	12 (28.6) 30 (71.4)	3.674 (0.055)
Family experience of will for organ donation	Yes No	1 (2.3) 42 (97.7)	3 (7.1) 39 (92.9)	1.099 (0.294)
Contact experience HSCD promotion	Yes No	16 (37.2) 27 (62.8)	8 (19.0) 34 (81.0)	3.459 (0.063)
Interest in HSCD	Yes	18 (42.9)	17 (40.5)	0.049
	No	24 (57.1)	25 (59.5)	(0.825)

Exp: Experimental group, Con: Control group.

**Table 3:** Homogeneity of the two groups based on general characteristics.

	Range of Score	Exp. (*n* = 43)	Con. (*n* = 42)	*t/Z*	*p*
Knowledge	0–1	0.40 ± 0.21	0.39 ± 0.22	0.173	0.863
Attitude ^†^	1–4	3.26 ± 0.39	3.14 ± 0.41	−1.456	0.145

Exp: Experimental group, Con: Control group, ^†^ Mann–Whitney test.

**Table 4:** Group differences in knowledge and attitude regarding HSCD.

	Knowledge			Attitude		
	Pre-test	Post-test	*F*^†^ (*p*)	Pre-test	Post-test	*F*^†^ (*p*)
Exp. (*n* = 43)	0.40 ± 0.21	0.76 ± 0.14	8.093 (<0.001)	3.26 ± 0.39	3.86 ± 0.37	−6.422 (<0.001)
Con. (*n* = 42)	0.39 ± 0.22	0.35 ± 0.22		3.14 ± 0.41	3.12 ± 0.35	

Exp: Experimental group, Con: Control group, ^†^ Analysis of covariance (ANCOVA; adjusting pre-test value).

**Table 5:** Group differences in willingness for HSCD.

Willingness for HSCD	Pre-Test			1st Post-Test		
	Exp. (*n* = 43)	Con. (*n* = 42)		Exp. (*n* = 43)	Con. (*n* = 42)	
	*n* (%)	*n* (%)	χ^2^ (*p*)	*n* (%)	*n* (%)	χ^2^ (*p*)
Yes	27 (62.8)	27 (64.3)	0.020	41 (95.3)	31 (73.8)	7.609
No	16 (37.2)	15 (35.7)	(0.886)	2 (4.7)	11 (26.2)	(0.006)

Exp: Experimental group, Con: Control group.

**Table 6:** Group differences in donor registration for HSCD.

Donor Registration to KONOS	2nd Post-test		
	Exp. (*n* = 43)	Con. (*n* = 42)	
	*n* (%)	*n* (%)	χ^2^ (*p*)
Yes	10 (23.3)	3 (7.1)	4.258
No	33 (76.7)	39 (92.9)	(0.039)

Exp: Experimental group, Con: Control group.
